# Supplementary figures and images for: A digital self-care intervention for Ugandan patients with heart failure and their clinicians: User-centred design and usability study
Source: Digit Health. 2022 Sep 27;8:20552076221129064. doi: 10.1177/20552076221129064 (PMC9520172; doi:10.1177/20552076221129064)

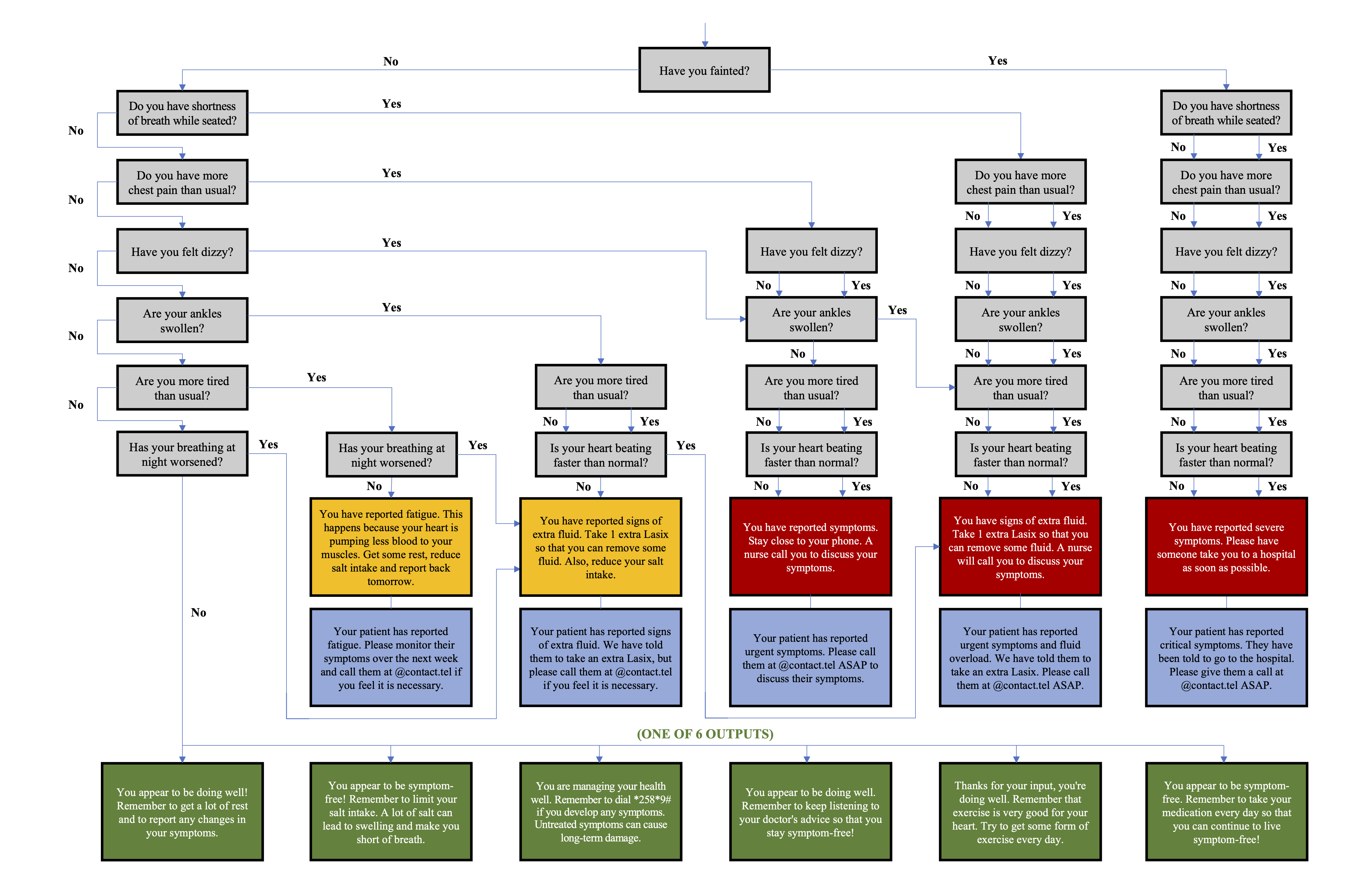

Supplement: sj-png-1-dhj-10.1177_20552076221129064 - Supplemental material for A digital self-care intervention for Ugandan patients with heart failure and their clinicians: User-centred design and usability study [file sj-png-1-dhj-10.1177_20552076221129064.png]
